# Systematic Functional Analysis of Bicaudal-D Serine Phosphorylation and Intragenic Suppression of a Female Sterile Allele of *BicD*

**DOI:** 10.1371/journal.pone.0004552

**Published:** 2009-02-23

**Authors:** Rafael Koch, Romana Ledermann, Olivier Urwyler, Manfred Heller, Beat Suter

**Affiliations:** 1 Institute of Cell Biology, University of Bern, Bern, Switzerland; 2 Department of Clinical Research, University of Bern, Bern, Switzerland; UT MD Anderson Cancer Center, United States of America

## Abstract

Protein phosphorylation is involved in posttranslational control of essentially all biological processes. Using mass spectrometry, recent analyses of whole phosphoproteomes led to the identification of numerous new phosphorylation sites. However, the function of most of these sites remained unknown. We chose the *Drosophila* Bicaudal-D protein to estimate the importance of individual phosphorylation events. Being involved in different cellular processes, *BicD* is required for oocyte determination, for RNA transport during oogenesis and embryogenesis, and for photoreceptor nuclei migration in the developing eye. The numerous roles of *BicD* and the available evidence for functional importance of BicD phosphorylation led us to identify eight phosphorylation sites of BicD, and we tested a total of 14 identified and suspected phosphoserine residues for their functional importance *in vivo* in flies. Surprisingly, all these serines turned out to be dispensable for providing sufficient basal BicD activity for normal growth and development. However, in a genetically sensitized background where the BicD^A40V^ protein variant provides only partial activity, serine 103 substitutions are not neutral anymore, but show surprising differences. The S103D substitution completely inactivates the protein, whereas S103A behaves neutral, and the S103F substitution, isolated in a genetic screen, restores *BicD^A40V^* function. Our results suggest that many BicD phosphorylation events may either be fortuitous or play a modulating function as shown for Ser^103^. Remarkably, amongst the *Drosophila* serines we found phosphorylated, Ser^103^ is the only one that is fully conserved in mammalian *BicD*.

## Introduction

Reversible phosphorylation of proteins at Ser, Thr and Tyr residues is a particularly important type of a posttranslational modification because it is involved in the control of essentially all biological processes. For this reason, protein phosphorylation has drawn widespread interest, and a number of techniques have been developed and were successfully applied to investigate the phosphorylation states and sites of isolated proteins. A combination of recent advancements in genomics and mass spectrometric analysis of peptides opened the possibility to analyze phosphorylation of whole proteomes, allowing the identification of many new phosphorylation sites (see e. g. [1]–[3]). However, the functions of these newly discovered phosphorylation events are usually not clear.

To estimate the importance of individual phosphorylation events, we set out to systematically verify the phosphorylation sites of one polypeptide, and to test these genetically for their functional importance. To increase the chances of identifying functional roles for these sites, we chose the *Drosophila* Bicaudal-D (BicD) protein that is phosphorylated [4] and has many essential functions during various phases of the life cycle of the fly [5]. In addition, there is evidence that the kinases *polo* [6] and *misshapen* [7] may phosphorylate *Drosophila* BicD, and the human Glycogen synthase kinase-3β (GSK-3β) can phosphorylate human BicD1 *in vitro* [8]. Furthermore, phenotypic correlations between phosphorylation and mutant phenotypes had already been described. The *BicD* allele *BicD^PA66^*, an alanine to valine substitution at amino acid 40, is a recessive hypomorphic mutant that is viable, but female sterile because their ovaries fail to differentiate an oocyte and egg. In these mutant ovaries, phosphorylation of the BicD^A40V^ protein is markedly reduced [4]. In addition, a suppressor of this allele, *BicD^PA66^ Su(66)*, restores female fertility as well as BicD phosphorylation levels [4]. *BicD* functions in numerous different processes, and we will therefore briefly summarize these functions to give an impression of the various tests we set up.

During early oogenesis, *BicD* is required for the determination and differentiation of an oocyte from a cluster of 16 interconnected germ cells. While the remaining 15 become nurse cells, the oocyte relocalizes in a *BicD*-dependent manner to the posterior of the developing egg chamber. In this process, *BicD* works together with *egl*, *Lis-1* and *Dynein* in a microtubule based process (reviewed in [5]), and the same machinery seems to function subsequently in delivering primary axis determination mRNAs (see e. g. [9]). Also during oogenesis, but as part of different processes with distinct requirements for additional genes, BicD localizes organelles and proteins to specific subcellular compartments [10]–[14]. During embryogenesis, the BicD-dependent RNA transport machinery is used again for the apical localization of pair-rule and *wingless* transcripts [15]–[17]. At the third instar larval stage, formation of the ommatidia of the compound eye starts and the nuclei of the differentiating photoreceptor cells migrate to the apical surface [18]. This apical migration is dependent on *BicD*, *Lis-1*, and the microtubule motors *Dynein* and *Kinesin* [7], [10], [19]. The highly regular geometry of the compound eye makes this a very sensitive system to study the effect of slight alterations of the activities of genes involved in its development. To systematically test the function of suspected and identified phosphorylation sites in BicD, we made mutants *in vitro* that cannot be phosphorylated at these sites (Ser to Ala or Asn substitutions) and mutants that mimic permanent phosphorylation of some of these sites (Ser to Asp). We then produced transgenic lines and crossed them into the *BicD^null^* mutant background [20] to test whether the mutant alleles were capable to substitute for the normal *BicD* in the various processes described.

Surprisingly, these phosphosites turned out not to be essential for any of the described *BicD* functions, suggesting that they are either redundant, only modulating or even fortuitous events. While limited tests for redundant functions also failed to uncover such events, one site turned out to be important for overall BicD phosphorylation levels. However, this mutant did still not reveal any other *BicD* phenotype, further arguing against essential functions of BicD phosphorylation in normal development. With the help of a genetically identified suppressor mutation that rescues BicD^A40V^ hypophosphorylation, we finally found evidence for a modulating role of Ser^103^. In the background of the only partially functional BicD^A40V^ variant, the side chain of position 103 becomes crucial for BicD function, even though it is not in the wild type background. While a S103A substitution does not change the BicD^A40V^ phenotype, the permanent phospho-mimic mutant S103D fully inactivates the protein, and a S103F mutant restores its activity significantly. If an extrapolation from our results is valid, most phosphorylation events may be fortuitous and play only a modulating role if any.

## Results

### Multiple phosphorylation sites in BicD

**Figure 1:**
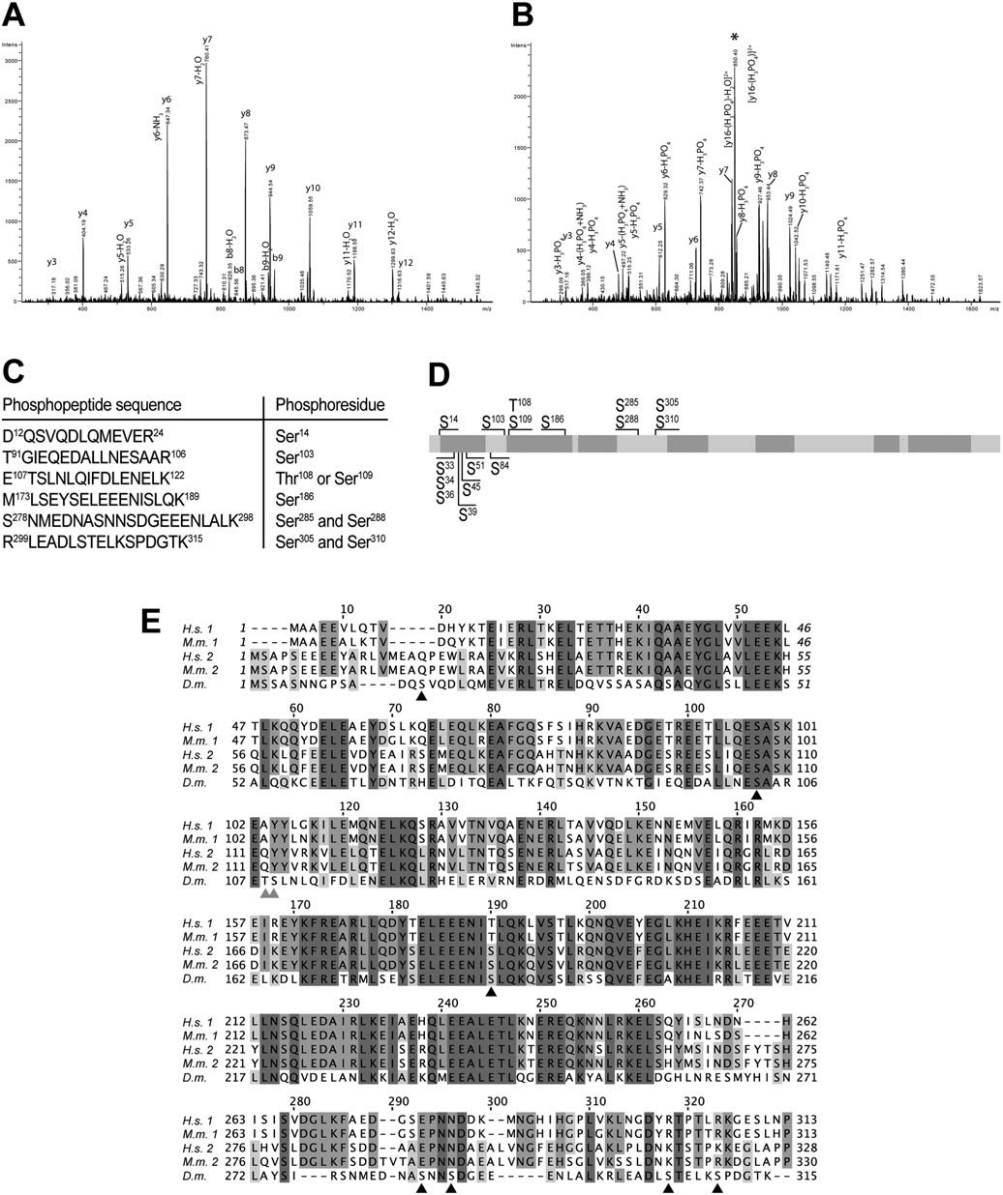
Location of BicD phosphorylation sites. A, B: MS/MS spectra of the [M+2H]^2+^ ions of the peptide T^91^GIEQEDALLNESAAR^106^ (A) and its serine phosphorylated form (B). The intense, neutral loss fragment at m/z = 850.4 (marked with an asterisk) in B indicates the extensive loss of phosphoric acid. Upon collision induced fragmentation in the iontrap, peptide bond fragmentation allowed unambiguous characterization of the amino acid sequence and the presence of a phosphorylated Ser. Note the m/z shift of 80 mass units corresponding to the phosphorylation of serine at y(4) and following y- ions between A and B. Furthermore, y-ions showed also extensive loss of phosphoric acid corresponding to a y-ion series with 98 mass units difference in the same MS/MS spectrum in B. C: Summary of phosphopeptides and phosphorylation sites of BicD identified by MS analysis. Phosphorylation of Ser^285^ was only observed when Ser^288^ was phosphorylated as well. Of Ser^305^ and Ser^310^, both, single and double phosphorylations, were found. The peptide 12–24 is an incomplete tryptic fragment, whereas the shown peptide 299–315 has two missed cleavage sites. Due to its small size, the peptide S^310^PDGTK^315^ could not be identified individually. D: Schematic drawing of the BicD protein. The positions of phosphoserines identified by MS analysis are indicated on top. Additional mutants created for this study are indicated at the bottom. Coiled-coil domains were predicted using the program MARCOIL [43], and are shaded in dark grey (probability ≥90%). Drawing is to scale. E: Amino acid alignment of the BicD N-terminal part. The sequences of human BicD1 and 2 (H.s. 1, H.s. 2; SwissProt entries Q96G01 and Q8TD16, respectively), mouse BicD1 and 2 (M.m. 1, M.m. 2; Q8BR07, Q921C5) and *Drosophila* BicD (D.m.; P16568) were aligned using the program T-COFFEE [44]. Phosphorylated serines in *Drosophila* BicD are marked by black triangles, ambiguous sites are indicated by grey triangles.

An initial analysis of BicD phosphorylation using *in vivo* ^32^P phosphate labeled ovaries combined with phospho-amino acid analysis revealed only significant phosphoserine signal, indicating that phosphorylation of ovarian BicD takes place preferentially at serines. CNBr mapping data further indicated that these phosphoserines are primarily present in the N-terminal region (peptide 21–138; S. Larochelle and B. Suter, personal communication). To identify BicD phosphorylation sites, we immunoprecipitated unlabeled protein from ovarian and embryonic extracts. Bands corresponding to BicD were excised from the gel and analyzed by mass spectrometry. Alternatively, BicD::GFP [21] was immunoprecipitated from embryo extracts with anti-GFP antibodies coupled to beads and analyzed by MS without a gel purification step. Phosphopeptides were subjected to tandem MS analysis to identify phosphorylated residues, as shown exemplarily for the peptide T^91^-R^106^ in Figure 1A and B (phosphorylated). The obtained data allowed unambiguous identification of phosphorylated serines at Ser^14^, Ser^103^, Ser^186^, Ser^305^ and Ser^310^. The latter two, Ser^305^ and Ser^310^, were also found to be simultaneously phosphorylated, as revealed by the identification of the doubly phosphorylated peptide R^299^/L^300^EADLpSTELKpSPDGTK^315^ with one or two missed cleavage sites. In addition, we found Ser^288^ to be phosphorylated, either alone, or together with Ser^285^, while we did not see Ser^285^ phosphorylation independently of pSer^288^. Moreover, either Thr^108^ or Ser^109^ become phosphorylated, but the lack of discriminative product ions did not allow unambiguous allocation of the phosphorylation site. Figure 1C summarizes the phosphorylation sites we identified. Phosphorylation of threonine or tyrosine was not observed in the MS analysis, which is in agreement with the earlier *in vivo* labeling result.

MS-identified phosphorylation sites are all located within the N-terminal half of BicD (Figure 1D). Remarkably, the Ser^103^ and Ser^186^ are conserved in human and mouse BicD, and they are situated each in regions that are highly conserved between fly, human and mouse BicD isoforms (Figure 1E), though S^186^ is substituted by a Thr in BicD1. All other serines, 14, 109, 285/288 and 305/310 are not conserved however, although Ser^109^ corresponds to a phosphorylatable tyrosine in mammalian BicD (Figure 1E). Evolutionary conservation is only one indication of functional importance and for Ser^14^ there is additional evidence. The sequence around Ser^14^ is a perfect match to a Polo kinase target site according to the consensus sequence D/E-X-S/T-Φ-X-D/E [22], where Φ denotes any hydrophobic residue. A Polo kinase target site is of particular interest because over-expression of Polo kinase in the germ line of *BicD^PA66^* females was reported to restore oocyte formation in most egg chambers and also normal distribution of the *BicD^PA66^* protein [6]. Surprisingly, even though this *PA66* mutation (A40V) affects overall BicD phosphorylation drastically [4] and the region around this substitution contains five serines between positions 33 and 45, none of them show any detectable Ser phosphorylation (Table 1). This was also unexpected because a similar Ser/Ala-rich region had been identified to be phosphorylated [23]. This finding indicates that these sites are either not phosphorylated or that these modifications are only short-lived.

**Table 1:** Phenotypes of *BicD* phosphorylation mutants.

Mutation	MS identification		Phenotype
	phos.	non-phos.	
wild type			ff
S14A	+	−	ff
S14D			ff
S33A	−	+	ff
S34A	−	+	ff
S36A	−	+	ff
S39A	−	+	ff
S45A	−	+	ff
multiple			ff
S51A	−	−	ff
S84A	−	−	ff
S103A	+	+	ff
S103D			ff
S103F			ff
S109A	+	+	ff
S186A	+	+	ff
S285A	+	+	ff
S288A	+	+	ff
S285A+S288A			ff
S305	+	+	ff
S310A	+	+	ff
S310D			ff
S305A+S310A			ff
A40V			fs, 16 nurse cells
A40V+S103A			fs, 16 nurse cells
A40V+S103D			lethal with few adults, 16 nurse cells
A40V+S103F			ff, few egg chambers with 16 nurse cells

Peptides containing the indicated serines were identified (+) as serine phosphorylated form (phos.) or non-phosphorylated (non-phos.), or the peptide was never identified (−). All mutants are viable, except for *BicD^A40V, S103D^*. *BicD^multiple^* consists of the five substitutions S33A, S34N, S36N, S39A, S45A.

ff females fertile.

fs females sterile.

### Testing the functional importance of BicD phosphorylation

To investigate the importance of phosphorylation for BicD function, a systematic mutagenesis study was carried out. Mutations changing the identified and candidate phosphorylation target sites were introduced by site directed mutagenesis into a functional, untagged *mini-BicD* gene, that is expressed from its native promoter and is also used as the wild type control gene (*BicD^wt^*; see Materials and Methods section for details). Accordingly, we made phosphorylation-impaired mutants for the serines shown in Figure 1C by substituting the respective codons individually with alanine codons. To investigate the possibility that transient phosphorylation of the serines in the vicinity of the *PA66* mutation (serines 33, 34, 36, 39, 45, 51 and 84) plays a functional role, we also tested mutations that change these Ser into Ala. At the same time we also tested for functional redundancy between sites in the serine 33–45 cluster. For this we made a quintuple mutation in which these Ser were replaced with Ala or Asn codons. For Ser^14^, Ser^103^ and Ser^310^, we additionally made phospho-mimic aspartic acid substitutions. Transgenic fly lines were established for all mutants and the constructs were crossed into hemizygous *BicD^null^* flies to test for their ability to restore viability and female fertility of the *BicD^null^* mutants. In this assay, the transgenic *BicD^wt^* construct was able to completely rescue viability and fertility of the null mutants, while a female sterile allele *BicD^PA66^*, reconstructed in the same mini gene (*BicD^A40V^*), produces viable but sterile females. Therefore, the *mini-BicD* rescue constructs show the same effects as the endogenous alleles and the assay system is thus validated.

Table 1 lists all mutations that were analyzed in this study and the associated phenotypes. Surprisingly, all Ser mutant constructs were able to rescue the lethality and the sterility of the *BicD^null^* allele, indicating that phosphorylation of these individual serines is not absolutely required to provide basal levels of essential *BicD* functions, and that the function of *BicD* in oogenesis cannot be dramatically impaired by any of these mutants. In addition, mimicking permanent phosphorylation of Ser^14^, Ser^103^ and Ser^310^ also appears not to have a major effect on *BicD* function.

**Figure 2:**
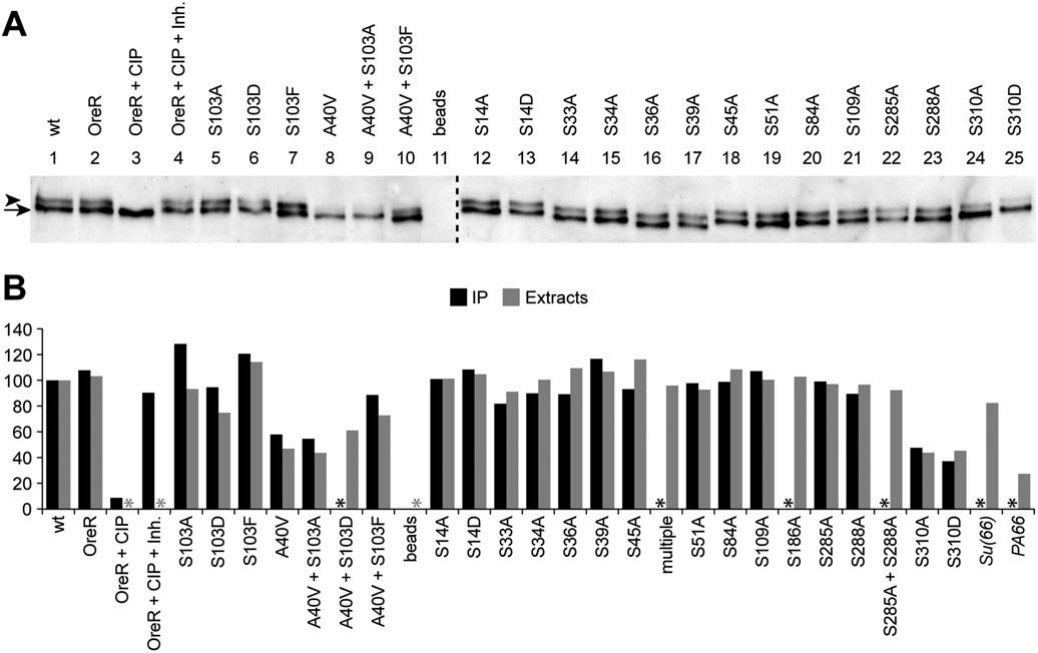
BicD phosphorylation in phosphorylation target site mutants. BicD protein isoforms were separated by PAGE and detected by Western blotting. A: A representative blot with BicD protein immunoprecipitated from the indicated ovarian extracts. All samples were processed simultaneously. The slowest migrating isoform corresponds to hyperphosphorylated BicD (marked with an arrowhead), as demonstrated by treatment of immunoprecipitates from OregonR females without (OreR) and with Phosphatase (+CIP), or with Phosphatase in presence of inhibitors (+CIP +Inh.). In addition, the reconstruction of the *BicD^PA66^* mutant (A40V) served as internal control, where the slow migrating isoform is reduced. Beads: mock IP from OreR extracts without antibody. The dashed vertical line indicates the border between different gels/blots. Due to the small ovary size of the female sterile mutants, less total material was loaded in lanes 8 and 9. B: For quantification, the amount of phospho-BicD was determined relative to the not mutated rescue construct (wt) that was set to 100%. All values are normalized to the sum of both bands to compensate for different total amounts loaded. Black columns: percentage of phospho-BicD determined from immunoprecipitation and the blot shown in panel A. Grey columns: percentage of phospho-BicD obtained from ovarian extracts without IP. *BicD^multiple^* consists of the five substitutions S33A, S34N, S36N, S39A, S45A. *Su(66)*: extracts from homozygous *BicD^PA66^ Su(66) cn* females. *PA66*: extracts from homozygous *BicD^PA66^ cn bw* females. *: no data.

Wild type BicD preferentially accumulates in the oocyte, and a significant amount of the protein is hyperphosphorylated. In *BicD^PA66^* mutants however, no oocyte is formed and the levels of hyperphosphorylated BicD are reduced [4]. To further test the correlation between *BicD* function and the presence of the hyperphosphorylated isoform of BicD, we analyzed the BicD isoform pattern in the newly established phosphomutants. For this purpose, BicD was immunoprecipitated from wt and mutant ovary extracts and its isoforms were separated by gel electrophoresis and visualized by western blotting. In control extracts from rescued wild type and OregonR females, there were two distinct isoforms of BicD present (Figure 2A, lanes 1 and 2, respectively). The BicD-antibody complex immunoprecipitated from OreR extracts was additionally treated with Calf Intestinal Phosphatase (CIP), which led to the disappearance of the slow migrating isoform (lane 3). Treatment in the presence of phosphatase inhibitors did not change the isoform pattern (lane 4), confirming that the slow migrating band is indeed a hyperphosphorylated isoform of BicD. Consistent with its normal physiological function, the isoform pattern of the wild type rescue construct was comparable to the one of the OreR controls (lanes 1 and 2), while the reconstructed *BicD^PA66^* mutant lead to a significant reduction of the slowest migrating band (lane 8), as had been reported for endogenous BicD^A40V^ [4].

Mutating the serines 14 through 288 has little effect on the global phosphorylation levels of BicD, and the patterns and the individual band intensities of BicD in these mutants appear similar to the wild type controls (Figure 2A, lanes 5, 6 and 12–23). Interestingly, in the case of Ser^14^ and Ser^103^, neither mimicking permanent phosphorylation nor preventing phosphorylation influences overall phosphorylation levels of BicD (Figure 2B and 2A, lanes 12, 13 and 5, 6, respectively). To further verify this visual impression, we quantitatively assayed the amount of phospho-BicD in these mutants. The graph in Figure 2B shows the amount of the phospho-isoforms of every mutant relative to the wild type rescue construct that was set to 100% (black columns). Because we could not see a major change of BicD protein content in any of the Ser mutants, we assumed that reduced hyperphosphorylation of BicD leads to a higher level of the hypophosphorylated isoform, rather than to degradation. Accordingly, the values of hyperphospho-BicD were normalized to the sum of both BicD bands in each individual sample. The quantification results confirmed the visual impression and revealed only minor differences in hyperphosphorylation of BicD between most mutants. We also repeated this phospho-BicD analysis with loading ovarian extracts directly on the gel and obtained the same results (Figure 2B, grey columns), indicating that the ratios are not distorted by differential immunoprecipitation of different isoforms.

While these findings are in agreement with a positive correlation between phospho-BicD levels and *BicD* function, the Ser^310^ mutants seems to be an exception to this. They are viable and normally fertile, but show a markedly reduced amount of hyperphosphorylated BicD, irrespective of whether phosphorylation of this residue is prevented or mimicked (Figure 2B and 2A, lanes 24 and 25). Because the Ser^310^ alleles are functional, while A40V is not, it is unlikely that the cause of the malfunction of the A40V mutation is solely reduced BicD phosphorylation.

*BicD* has additional zygotic functions later in development, where it is required for the positioning of the photoreceptor nuclei [10]. Analyzing the eyes of *BicD^null^* flies reveals a rough eye phenotype with irregularly shaped ommatidia (data not shown; see later). To test whether one of our serine mutants plays a role specifically in this pathway, we inspected the eyes of these mutants and found them to be normal, suggesting that BicD phosphorylation is also dispensable for this process (data not shown).

### Substituting Ser^103^ by phenylalanine in BicD^A40V^ suppresses the *BicD^PA66^* phenotype and increases phospho-BicD levels

While phosphorylation of the 14 tested serines is not essential for basal BicD function, it may play a more modulating role that can be detected under less favorable conditions. A genetic screen for a suppressor of the phosphorylation mutant *BicD^PA66^* lead to the isolation of the *Su(66)* mutation that significantly restores the accumulation of hyperphosphorylated BicD protein [4]. In addition, the suppressor mutation also restores female fertility and oocyte localization of BicD. As these observations point to a functional importance of BicD phosphorylation, we set out to study the molecular basis of this phenotype. The *Su(66)* mutation maps to the second chromosome and recombination mapping experiments placed *Su(66)* in the immediate vicinity of *BicD* (A. Swan and B. Suter, personal communication). In order to identify this suppressor mutation, we sequenced *BicD* and its four proximal neighboring genes *Sgt*, *Aac11*, *fws* and *CG5110* from homozygous *BicD^PA66^ Su(66)* flies. The sequences were compared to the parental *BicD^PA66^* chromosome. No polymorphism was detected in the four proximal genes and the *BicD^PA66^* mutation was present on the *BicD^PA66^ Su(66)* chromosome as expected. In addition, we found in the *BicD* gene a single nucleotide transition C→T that was not present in the parental *BicD^PA66^* strain. This mutation changes the codon 103 from TCC to TTC, causing the normally present serine to be substituted by a phenylalanine in *Su(66)*. This substitution was of exceptional interest, because our MS analysis identified this Ser^103^ to be phosphorylated. In order to test whether the S103F substitution indeed acts as suppressor of the *BicD^PA66^* allele, we reconstructed this *BicD* allele with both mutations. Indeed, females with one copy of this double mutant chromosome *BicD^A40V, S103F^* were viable and fertile, while the ones with *BicD^A40V^* alone are viable but sterile.

**Figure 3:**
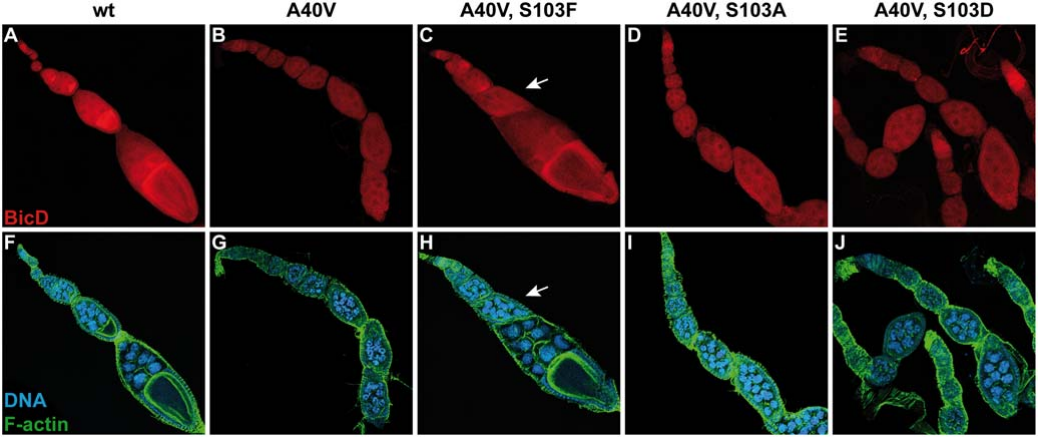
A S103F substitution in *BicD^A40V^* suppresses the *BicD^PA66^* phenotype. Confocal images of ovaries from the indicated wt and mutant females, stained with anti-BicD antibodies (red). Blue: DNA, green: F-actin. A, F: In ovaries with wild type *BicD*, all egg chambers contain an oocyte, and the protein accumulates in the oocyte throughout oogenesis. The *BicD* mutants A40V (B, G) fail to form an oocyte, and egg chambers contain 16 nurse cells. The mutant protein does not accumulate in a single cell. C, H: Substitution of Ser^103^ by phenylalanine in *BicD^A40V^* suppresses the *BicD^PA66^* phenotype. Most egg chambers form an oocyte, where the BicD protein accumulates to a certain extent. The egg chamber indicated by an arrow contains 16 polyploid nurse cells and no oocyte. D, I: The mutants A40V+S103A and A40V+S103D (E, J) also fail to form an oocyte. Shown are maximum projections of z-stacks. Panels F–J: for clarity reasons, individual panels are composed of different optical sections.

In order to study the effects of this mutation, we analyzed the influence of residue 103 on the distribution of the protein during oogenesis. At first glance, ovaries of *BicD^A40V, S103F^* flies appear largely normal and contain mostly egg chambers with normal morphology (Figure 3). The mutant BicD protein accumulates in the oocyte and displays a normal subcellular distribution. However, the accumulation appears less pronounced compared to the wild type situation (Figure 3A, F), suggesting that the double mutant BicD protein is less active than wild type BicD. Moreover, such BicD^A40V, S103F^ ovaries contain a few egg chambers that failed to form an oocyte, and, instead, contain 16 polyploid nurse cells (arrow in Figure 3C, H), like all egg chambers from control *BicD^A40V^* females do (Figure 3B, G). This is consistent with our previous observations [4], confirming that the S103F substitution is sufficient to partially suppress the effects of the *BicD^A40V^* mutation.

### Ser^103^ substitutions alter the *BicD^A40V^* phenotype in a mutant specific manner

Because phenylalanine is not phosphorylatable, we wondered whether preventing Ser^103^ phosphorylation is sufficient to suppress the *BicD^PA66^* phenotype. To test this, we constructed the *BicD^A40V, S103A^* allele, where the Ser^103^ is replaced by an alanine, which cannot be phosphorylated either. Surprisingly however, such *BicD^A40V, S103A^* females were sterile with ovaries consisting of egg chambers with 16 nurse cells and no oocyte (Figure 3D, I), indistinguishable from the phenotype of *BicD^A40V^* females that have the wild type serine at position 103. Therefore, the suppression effect of the S103F substitution on BicD^A40V^ cannot be caused simply by inhibition of phosphorylation of Ser^103^.

**Figure 4:**
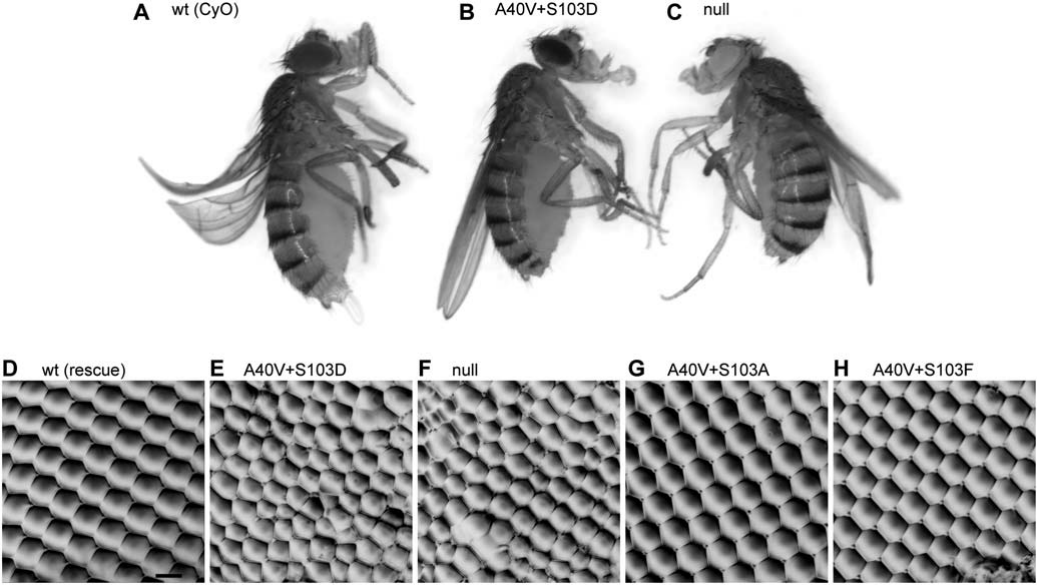
The *BicD^A40V, S103D^* mutant phenocopies the *BicD^null^* allele. A normally sized fly with one copy of wild type BicD from the CyO chromosome (A) compared with smaller *BicD^A40V, S103D^* (B) and *BicD^null^* (C) siblings. Note that these two mutants also display short sternopleural and scutellar bristles. Panels D–H: Nail polish imprints of eyes of adult *BicD* mutants. D: rescue *BicD^wt^*. E: *BicD^A40V, S103D^*. F: *BicD^null^*. G: *BicD^A40V, S103A^*. H: *BicD^A40V, S103F^*. The black bar in panel D indicates 15 µm.

We next wondered how mimicking permanent phosphorylation of Ser^103^ in BicD^A40V^ affects the function of the protein. Strikingly, *BicD* with both substitutions, A40V and S103D, does not rescue *BicD^null^* alleles and thus behaves like a recessive lethal mutant. Only very few *BicD^A40V, S103D^* adults were obtained, they hatched 2–4 days later and were smaller than control siblings with an endogenous copy of wild type *BicD* from the *CyO* chromosome (Figure 4A–C), appearing weak and lethargic and displaying uncoordinated behavior. These mutants died within a few days. In addition, they had a variable rough eye phenotype because of irregular ommatidia (compare Figure 4D–F), while the viable *BicD^A40V, S103A^* and *BicD^A40V, S103F^* mutants displayed normal eyes (Figure 4G and H, respectively). Ovaries of *BicD^A40V, S103D^* females consist of egg chambers lacking an oocyte and containing only 16 polyploid nurse cells (Figure 3E and J), similar to ovaries from *BicD^PA66^* and *BicD^null^* flies (Figure 3B, and [20], [24]). In addition, the BicD^A40V, S103D^ protein does not accumulate into a single cell. All these described phenotypes were previously reported for *BicD^null^* flies [10], [20]. These findings therefore strongly suggest that mimicking permanent phosphorylation at amino acid position 103 of BicD^A40V^ severely inhibits even the essential zygotic functions of BicD^A40V^.

### Effect of amino acid 103 on BicD function

In an otherwise wild type BicD, substituting the Ser^103^ by A, D or F does not yield any obvious phenotypes. We were therefore surprised by the strikingly different phenotypes observed when these substitutions were combined with the A40V mutation found in *BicD^PA66^*. Consequently, we also analyzed the function of residue 103 in the wild type protein in more detail. The S103A/D/F mutants were viable and fertile (Table 1). However, the phospho-mimic *BicD^S103D^* mutant seems to have slightly less hyperphosphorylated BicD than the phosphorylation defective mutants *BicD^S103F^* and *BicD^S103A^* (Figure 2B and 2A, lanes 5–7). Ovaries of *BicD^S103X^* females appear normal, and while the S103A mutant protein accumulates normally in the oocyte, the BicD^S103D^ protein shows somewhat reduced and the S103F protein slightly increased oocyte accumulation (Supporting Figure S1). These findings suggest that mimicking permanent phosphorylation of BicD at Ser^103^ acts inhibitory on the protein's oocyte localization.

**Figure 5:**
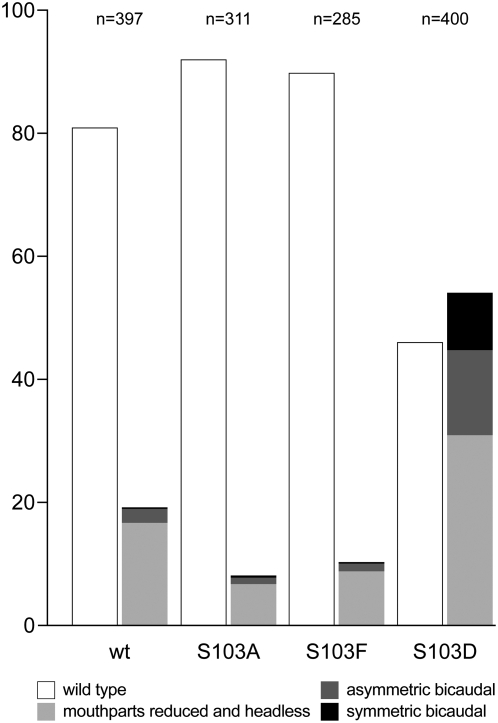
Substitutions in amino acid 103 of BicD modulate the dominant *BicD^2^* phenotype. Embryos were collected from mothers hemizygous the dominant *BicD^2^* allele, and carrying one copy of transgenic *BicD^wt^*, *BicD^S103A^*, *BicD^S103D^*, or *BicD^S103F^*. The embryos were scored for anterior defects and classified according to the denoted categories. N: total number of embryos counted. The flies and the embryos were raised at 25°C and shifted to 18°C one day before egg collection.

Another assay to test the activity of the Ser^103^ mutants is to investigate whether these mutants affect the dominant *BicD* phenotype. Females with the *BicD^2^* allele produce embryos with defective anterior structures [24], [25] caused by a partial mislocalization of *osk* mRNA to the anterior of the oocyte and the embryo [26], [27]. Embryos from females hemizygous for *BicD^2^* and with one copy of the wild type rescue transgene (*BicD^wt^*) or a Ser^103^ mutant construct were inspected for defective anterior structures (Figure 5). With the *BicD^wt^* transgene, such mothers produce mostly normal embryos (wt). Similarly, low numbers of aberrant embryos are observed when the mothers had the S103A or S103F substitution in the *BicD* transgene. In contrast, a markedly increased amount of defective embryos was found when mothers carried the *BicD^S103D^* allele. These results provide further evidence that the amino acid at position 103 is important for full *BicD* function, and they suggest that transient phosphorylation of the native serine at this position plays a role in modulating *BicD* function.

## Discussion

Protein phosphorylation is a posttranslational modification that is used to regulate the function of proteins involved in many different cellular processes. The widespread interest in this reversible protein modification recently led to the analysis of phosphoproteomes (see e. g. [28], [29]) which revealed many new phosphorylation sites. However, the function of these phosphorylation events usually remained to be elucidated. To obtain an estimate of the function of the numerous phosphorylation sites determined with this technique, we picked a protein that is known to be phosphorylated, determined its phosphorylation sites, compared these sites with the proteomics data, analyzed the evolutionary conservation of the sites, and tested the requirement for phosphorylation of these residues. Our choice of example protein was the *Drosophila* BicD because it is conserved up to humans, is involved in different cellular processes that act during different phases of the life cycle, and because null mutants are available that allow us to test the functions of phosphorylation in all these phases. Using mass spectrometric analysis of immunoprecipitated BicD and BicD::GFP, we identified the serines 14, 103, 186, 285, 288, 305 and 310, and either Thr^108^ or Ser^109^ to be phosphorylated. Some of the sites found here have been determined independently by large scale screens for phospho-sites in *Drosophila* Kc167 cells [30] and in *Drosophila* embryos [31].

To find out more on the function of the BicD phosphorylation sites, we performed *in silico* analysis on them. Only three of the experimentally identified BicD phosphosites were also predicted with a bioinformatic analysis using the programs NetPhos [32] and Scansite [33]. The three broad phosphorylation motif classifications each fit one of the BicD phsophoserines. Ser^109^ corresponds to a basophilic site (K/R-X-X-S/T), while Ser^288^ fits an acidophilic motif (S/T-X-X-D/E), and Ser^310^ corresponds to a proline directed site (S/T-P). Very recently, a study on mitotic phosphorylation identified the unique phosphorylation motif S-G/A-X-K/R [29]. While the kinase for this site is yet unknown, the Ser^103^ matches this consensus phosphorylation motif.

In addition, we found that Ser^14^ is a perfect match to a Polo kinase target site D/E-X-S/T-Φ-X-D/E [22]. However, neither the S14A nor the S14D substitution exhibit an obvious phenotype, suggesting that phosphorylation of this residue is not required for proper *BicD* function. This finding is surprising, because a recent report suggested *polo* kinase to be involved in polarized transport during oogenesis, where Polo could activate BicD by phosphorylation specifically during oocyte differentiation [6]. Recently, the human Glycogen synthase kinase-3β (GSK-3β) was reported to form complexes with human BicD1 in a kinase activity-dependent manner [8], but the phosphorylated serines identified in this study (Figure 1E) do not fit the known GSK-3β consensus sequence S/T-X-X-X-S/T [34].

Our systematic *in vivo* analysis of BicD phosphorylation mutants revealed that none of these eight phosphorylation sites is essential for any BicD function, and that, with the exception of the Ser^310^ substitutions, global BicD phosphorylation levels remain unchanged in the tested mutants. One explanation for this could be that the hyperphosphorylated isoform contains multiple phosphorylation events between Ser^14^ and Ser^288^ and that the absence of a single one of them does not alter the isoform mobility. The exception is the serine 310. Surprisingly, BicD phosphorylation is markedly reduced in both, the S310A mutant that abolishes phosphorylation and the phospho-mimic S310D mutant. Even though S310 is crucial for overall BicD phosphorylation levels, this seems not to affect BicD activity much, as Ser^310^ mutants appear normal, further arguing against critical roles of BicD phosphorylation on its activity. In contrast, the A40V substitution that shows a similar reduction of total BicD phosphorylation, also greatly reduces the functionality of BicD. This suggests that the loss of phosphorylation in this mutant is a side effect or a consequence, rather than the cause of the loss-of-function, and that the bulkier side chain of valine causes a structural change in the mutant protein and that this inactivates the protein directly. Limited redundancy tests showed that in the case of the serines 285/288, and 305/310, which we found to be doubly phosphorylated, neither site is required for BicD function (Table 1). Similarly, the five serines 33–45 in the region around the A40V mutation are also dispensable for essential BicD activity.

Interestingly, it was a genetic screen for a suppressor of the female sterile and partially phosphorylation defective *BicD^A40V^* mutant that lead to the isolation of the *Su(66)* mutant that revealed the only identifiable function of BicD phosphorylation. We identified this mutation as a S103F substitution in the *BicD^A40V^* background and we showed that this substitution is sufficient to restore the critical functions of BicD. The analysis of additional Ser^103^ substitutions in the *BicD^A40V^* background provided further evidence that Ser^103^, and possibly its phosphorylation, play a modulating role on BicD function. Strikingly, these mutants exhibited very different phenotypes: while the S103A substitution does not change the *PA66* phenotype, the S103D mutation inactivates the BicD^A40V^ protein, causing *BicD^A40V, S103D^* to behave as a null mutant. The suppression effect of the S103F mutation in *Su(66)* cannot be caused by the absence of phosphorylation of Ser^103^, because the S103A substitution is neutral and does not change the *PA66* phenotype. Instead, the results suggest that the bulky side chain of phenylalanine introduces a structural change in the mutant protein that compensates for the loss of function caused by the A40V mutation. How this substitution can suppress over the relatively large distance of some 60 amino acids in the primary structure is presently unknown. However, Ser^103^ is located at the beginning of a predicted coiled-coil motif of BicD (Figure 1D), and it is tempting to assume that phosphorylation of Ser^103^ may regulate the formation of this coiled-coil region. Unfortunately, an earlier functional analysis of coiled coil regions of BicD did not test the importance of this particular coiled coil domain and only included one of the phosphoserines, S186 [35].

At the onset of our experiments we envisioned that individual phosphorylation events may control individual localization processes. The fact that all the biochemically identified phosphosites are non-essential for normal *BicD* function in all the different processes examined, however, suggests that most of the phosphorylation events are either fortuitous or play only minor modulating roles. The only modulating role we could find so far is one that we identified with tools created by a classical genetic approach, isolating a suppressor mutation that can restore the activity of a partially inactivated and hypophosphorylated variant of BicD. In this genetically sensitized background, the 103 position that gets phosphorylated in the wild type protein becomes decisive for the function of the entire protein. Interestingly, this Ser is also the only one that is fully conserved in mice and humans. According to our findings on BicD phosphorylation, the identification of protein phosphorylation sites needs to be treated with caution as such sites are often not crucial for the function of a protein.

## Materials and Methods

### Isolation of genomic DNA and sequencing of the *Su(66)* region

DNA from a pool of 15 flies was isolated according to [36]. The coding sequences of *BicD* and four proximal neighboring genes were amplified by PCR, skipping 7 kb of the first, large *BicD* intron. All PCR products were purified and sequenced on an ABI Prism 3100 Sequence Analyzer (Applied Biosystems).

### Generation of vectors, *in vitro* mutagenesis and transgenesis

We constructed the *pw+SNattB* vector that is designed to harbor constructs driven by their own promoter. *pw+SNattB* contains a multiple cloning site, a *white^+^* selectable marker, a *loxP* site, and an *attB* fragment that allows its integration into *attP* landing platforms [37] using the phiC31 integrase. Briefly, the *UAS-SV40* cassette from *pUASTattB* [37] was replaced with a modified multiple cloning site of *pLitmus28* (New England Biolabs) to yield *pw+SNattB*. The sequence is available from the EMBL/GenBank data libraries under accession no. EU729722. A similar strategy was used to construct *pUAS-K10attB* that is useful for gene expression in the female germline using the UAS-Gal4 system. The *UAS-SV40* cassette was removed from *pUASTattB* [37] and replaced with the *UASp* cassette from *pUASp* [38] to yield *pUAS-K10attB*. The sequence is available from the EMBL/GenBank data libraries under accession no. EU729723.

The *mini-BicD::GFP* fusion construct [21] was transferred as *Kpn*I/*Not*I fragment into *pw+SNattB*. The *BicD* 3′ part with the GFP fusion was then replaced with the corresponding native 2.75 kb *BicD* 3′ part lacking GFP that was taken from *pBS4.2RV3*′ [24]. This yielded the *mini-BicD-pw+SNattB* vector that served as wild type control *BicD^wt^*. A *BsiW*I/*Age*I fragment of *mini-BicD::GFP* was subcloned into *pLitmus28* (New England Biolabs), and the mutations were introduced in this construct by high fidelity PCR using suitable primers. Plasmids containing the correct mutations were further verified by sequencing. The individual mutations were then transferred into *mini-BicD-pw+SNattB* to replace the respective wild type sequence. The fragments containing the mutations S14A/D–S54A were cloned using *BsiW*I/*EcoR*I or *BsiW*I/*PshA*I, while S84A–S310A/D were transferred using *EcoR*I/*BstE*II or *PshA*I/*BstE*II. Most clones were error-free, however, a small number showed 1–3 nucleotide long deletions at the *PshA*I site in the intron 1.

These *BicD* mutants were introduced into *attP* landing platforms using the phiC31 integrase [37]. The mutants S33A, S34A, S36A, S45A, multiple, A40V, S103A/D/F, A40V+S103A/D/F and the wild type control were integrated into the landing platform ZH-102F on the fourth chromosome. S14A/D, S51A, S84A, S109A, S186A, S285A, S288A, S305A, S310A/D and the double mutants S285A/S288A and S305A/S310A were inserted into ZH-64A on the third chromosome, and S39A in ZH-2A on the X chromosome. All transgenic constructs in the flies were verified by preparation of genomic DNA and sequencing of the relevant regions containing the mutation(s).

### Fly stocks

The landing platforms and the integrases were described [37]. The *BicD^null^* allele *BicD^r5^* was described earlier [20], and *Df(2L)Exel7068* was obtained from Bloomington Stock Center (stock no. 7838). All *BicD* mutants generated for this study were kept as stocks with the *BicD^r5^* allele on the second chromosome (genotype *w*; *BicD^r5^ cn*/*SM1*; *+*; *+* with a *BicD* transgene on the 1^st^, 3^rd^ or 4^th^ chromosome as described above). Males were crossed to *w*; *Df(2L)Exel7068*/*CyO b* females as needed to generate flies carrying one copy of a *BicD* construct in a hemizygous *BicD^r5^* background. These progeny were then used for the experiments.

### Eye imprints and embryo cuticle preparations

The eye imprints were done as described in [39]. Images were recorded on a Leica DM6000 B microscope using a 40× DIC objective. For cuticle preparations, embryos were collected for 24 h at 18°C from *w*; *BicD^2^*/*BicD^r5^ cn*; *+*; *att-102F{BicD^mutant^}*/*+* mothers, with *BicD^mutant^* as indicated in Figure 5. The embryos were aged for 24 h at 25°C, and cuticles were prepared as described [40], mounted in a 1∶1 mixture of lactic acid∶Hoyer's medium and incubated at 58°C for 30 h. Cuticle phenotypes were scored on a Nikon Eclipse E600 microscope.

### Immunostainings

Immunohistochemical stainings were essentially done as described earlier [4], with denoted modifications. After fixation, ovaries were incubated in PBTM (1×PBS with 0.2% Tween-20, 0.1% Triton X-100 and 5% non-fat dry milk), and then with the appropriate antibodies in PBTM for 4 hours at room temperature. Mouse monoclonal anti-BicD antibodies 4C2 and 1B11 [4] were used each at 1∶15 dilution. The secondary Cy3-conjugated anti-mouse antibodies (Jackson ImmunoResearch) was used at a dilution of 1∶1,000. During the final washing steps, DNA and F-actin were stained with 2.5 µg/ml Hoechst 33258 (Molecular Probes) and 0.05 µg/ml FITC-conjugated phalloidine (Molecular Probes), respectively. The ovarioles were embedded in Aquamount (Polysciences), and images were recorded using a Leica DM6000 B fluorescence microscope or a Leica TCS-SP2 confocal microscope. To exclude artifacts resulting from small differences in buffers and incubation times, all samples were processed simultaneously, and images were recorded using identical settings on the microscope and the software.

### Large scale immunoprecipitations and mass spectrometry

Embryos were collected during 12 hour time periods, dechorionated and stored at −80°C. Ovaries from 10 g of flies were collected as described [41]. The egg chambers were washed twice with IP buffer (25 mM HEPES pH 7.4, 150 mM NaCl, 0.5 mM EDTA, 1 mM DTT, supplemented with complete protease inhibitors; Roche) and frozen in liquid nitrogen. In a glass homogenizer, 4 g of embryos or 3.5 ml of ovaries were homogenized in 8 ml (3.5 ml) IPpi buffer (IP buffer including phosphatase inhibitors 0.3 mM sodium orthovanadate, 2 mM sodium molybdate, 50 mM sodium fluoride). The homogenate was centrifuged for 1 h at 16,000 g at 4°C. The soluble phase was centrifuged again for 25 min at 16,000 g at 4°C. Two hundred µl GammaBind Plus Sepharose beads (GE Healthcare) were washed three times in PBS and incubated with 4 ml 1B11 anti-BicD antibody for two hours. Beads with bound antibodies were washed three times in PBS and once in IPpi buffer, added to the homogenate supernatant and incubated for 3 h at 4°C with constant mixing. The beads were then washed six times with IP buffer. Finally, the beads were resuspended in Nu-PAGE sample buffer (Invitrogen) containing 0.1 M DTT, boiled for 5 min, and proteins were separated by SDS PAGE. The gel was stained with Coomassie Blue (Invitrogen), bands of interest were excised, and proteins were digested in gel with sequencing grade trypsin (10 ng/ml; Promega) over night at room temperature. Peptides were extracted from the gel with 20% formic acid (FA) by incubation for 15 min at room temperature and analyzed by LC-MS/MS (Esquire3000+ ion trap mass spectrometer with a capillary ESI source (Bruker Daltonics) equipped with an Alliance HT2795 HPLC system from Waters). CID spectra interpretation was performed with the Phenyx software (GeneBio) using the Uniprot *Drosophila* protein database, release 54.0.

To immunoprecipitate BicD::GFP from embryonic extracts, anti-GFP antibodies were coupled covalently to Sepharose beads. One hundred µl GammaBind Plus Sepharose beads were washed three times in PBS and incubated with 2 ml anti-GFP antibody (mouse monoclonal 3E6, gift from A. Marcil, BRI, Montreal) for two hours. Beads with bound antibody were washed three times in PBS, and finally resuspended in 400 µl PBS. To this, 100 µl disuccinimidyl suberate solution (13 mg/ml in DMSO; Pierce Biotechnology) was added and incubated for 1 h with constant mixing. The beads were sedimented by centrifugation for 3 min at 1,300 g, washed once with 0.2 M ethanolamine pH 8.0 for 2 min and another time for 2 hours with constant mixing. Beads were sedimented and washed twice with 0.1 M glycine pH 2.8 for 10 min, and then 3 times 10 min with PBS. Finally, the beads were resuspended in RIPApi buffer (50 mM Tris-HCl pH 8.5, 300 mM NaCl, 0.1% SDS, 0.5% deoxycholate, 1% Nonidet-P40, 1 mM EDTA supplemented with protease and phosphatase inhibitors). Extracts of BicD::GFP embryos in RIPApi buffer were obtained as described above, and incubated with the anti-GFP beads over night at 4°C with constant mixing. The beads were washed on ice six times in RIPApi buffer and once for 3 min on ice in 0.1 M glycine pH 2.8. Beads were then sedimented by centrifugation, and bound proteins were eluted by incubation with 8 M urea/50 mM Tris-HCl pH 8.0 for 5 min at room temperature. The elution was repeated once, and the fractions were pooled. DTT was added to 5 mM, and the mixture was incubated at 37°C for 45 min. Sulfhydryl groups were derivatized for 30 min at 37°C in the dark by addition of 0.5 M iodoacetamide to 12.5 mM. The proteins were precipitated with acetone, resuspended and digested with trypsin (4 ng/ml) over night at room temperature. The digest was acidified with 20% FA and incubated with TiO_2_ slurry in 1.5× loading buffer (3.7 mg/ml TiO_2_ in 1.5% trifluoroacetic acid, 60% acetonitrile (MeCN), 1.5 M lactic acid) for 15 min. Beads were washed once in 1× loading buffer and twice with 5% MeCN. Bound peptides were eluted for 5 min with 50 mM phosphate, 5 mM sodium orthovanadate, 1 mM NaF at pH 10.5, acidified with 20% FA and dried in a vacuum centrifuge. Peptides were reconstituted in 25 µl 20% FA, and 20 µl were analyzed by nano-LC-MS/MS (LTQ-orbitrap-XL equipped with a nanospray probe and two Rheos micro/nanoflow HPLC systems; ThermoFisher Scientific). MS/MS spectra were searched with Phenyx software (GeneBio) against the Uniprot-Swissprot *Drosophila melanogaster* database, version 54.6. All identifications on phosphopeptides were manually validated for correctness.

### Small scale immunoprecipitations and western blotting

To prepare ovary extracts, ovaries from 1–2 day old females were dissected in *Drosophila* Ringer's solution (182 mM KCl, 46 mM NaCl, 3 mM CaCl_2_, 10 mM Tris-HCl, pH 7.2) and snap-frozen on dry ice. For every sample, 35 ovary pairs were used, except for *BicD^A40V^* and *BicD^A40V, S103A^*, where 70 pairs were used each, and OreR with 90 pairs. The ovaries were homogenized in 500 µl RIPApi buffer, and the homogenate was centrifuged twice for 5 min at 16,000 g at 4°C. The supernatant was combined with 30 µl of each anti-BicD antibody 1B11 and 4C2 and incubated for 2 h at 4°C with constant mixing. GammaBind Plus Sepharose beads were resuspended in RIPApi buffer, and 30 µl of this mixture was added to the ovary extracts and incubated for 1.5 h at 4°C with constant mixing. The beads were allowed to sediment by gravity and were washed 4 times with wash buffer 1 (RIPApi with only 0.5% Nonidet P-40).

For phosphatase treatment, beads were washed twice with wash buffer 1, once with wash buffer 2 (wash buffer 1 lacking phosphatase inhibitors) and once in a 1∶1 mixture of wash buffer 2 and NEB3 buffer (100 mM NaCl, 50 mM Tris-HCl, 10 mM MgCl_2_, 1 mM DTT, pH 7.9; New England Biolabs). The beads were split into 3 portions, resuspended in 30 µl NEB3 buffer, and incubated at 37°C for 1 h with 10 units of calf intestinal phosphatase (CIP; New England Biolabs). Controls were incubated without CIP, and with CIP in the presence of inhibitors (10 mM Na_3_VO_4_ and 4 mM Na_2_MoO_4_). The reaction was stopped by washing the beads 3 times with wash buffer 2 containing 1 mM EDTA. Finally, the beads were resuspended in 2× sample buffer (100 mM Tris-HCl pH 6.8, 20% glycerol, 4% SDS, 0.2 M DTT and a trace amount of bromophenol blue) and boiled for 5 min before being loaded on a gel.

To analyze ovary extracts without IP, ovaries were dissected and extracts were prepared as described [42]. Phospho-isoforms of BicD were separated on standard 8.5% polyacrylamide gels (Acrylamide∶Bis = 95∶1) lacking SDS, which was only provided in the running buffer. Gels were run at 20 mA in the stacking gel and at 38 mA in the separating gel with cooling to 15°C, using a Protean II xi cell (Bio-Rad). After transferring the proteins to nitrocellulose membranes, BicD was detected using the mouse anti-BicD antibodies 1B11 and 4C2 at a 1∶20 dilution each. Horseradish peroxidase-conjugated anti-mouse antibodies (GE Healthcare) were used at a dilution of 1∶5,000. The blots were probed with ECL plus reagents (GE Healthcare), and chemiluminescence signal was detected using a LAS-1000 detection system (Fujifilm). Western blots were evaluated using AIDA software (Raytest GmbH).

## Supporting Information

Figure S1 Effect of amino acid 103 on BicD localization
(0.57 MB PDF)

## References

[1] Bodenmiller B, Mueller LN, Mueller M, Domon B, Aebersold R (2007). Reproducible isolation of distinct, overlapping segments of the phosphoproteome.. Nat Methods.

[2] Collins MO, Yu L, Choudhary JS (2007). Analysis of protein phosphorylation on a proteome-scale.. Proteomics.

[3] Ficarro SB, McCleland ML, Stukenberg PT, Burke DJ, Ross MM (2002). Phosphoproteome analysis by mass spectrometry and its application to Saccharomyces cerevisiae.. Nat Biotechnol.

[4] Suter B, Steward R (1991). Requirement for phosphorylation and localization of the Bicaudal-D protein in Drosophila oocyte differentiation.. Cell.

[5] Claussen M, Suter B (2005). BicD-dependent localization processes: from Drosophilia development to human cell biology.. Ann Anat.

[6] Mirouse V, Formstecher E, Couderc JL (2006). Interaction between Polo and BicD proteins links oocyte determination and meiosis control in Drosophila.. Development.

[7] Houalla T, Hien Vuong D, Ruan W, Suter B, Rao Y (2005). The Ste20-like kinase misshapen functions together with Bicaudal-D and dynein in driving nuclear migration in the developing drosophila eye.. Mech Dev.

[8] Fumoto K, Hoogenraad CC, Kikuchi A (2006). GSK-3beta-regulated interaction of BICD with dynein is involved in microtubule anchorage at centrosome.. EMBO J.

[9] Clark A, Meignin C, Davis I (2007). A Dynein-dependent shortcut rapidly delivers axis determination transcripts into the Drosophila oocyte.. Development.

[10] Swan A, Nguyen T, Suter B (1999). Drosophila Lissencephaly-1 functions with Bic-D and dynein in oocyte determination and nuclear positioning.. Nat Cell Biol.

[11] Brendza RP, Serbus LR, Saxton WM, Duffy JB (2002). Posterior localization of dynein and dorsal-ventral axis formation depend on kinesin in Drosophila oocytes.. Curr Biol.

[12] Januschke J, Gervais L, Dass S, Kaltschmidt JA, Lopez-Schier H (2002). Polar transport in the Drosophila oocyte requires Dynein and Kinesin I cooperation.. Curr Biol.

[13] Duncan JE, Warrior R (2002). The cytoplasmic dynein and kinesin motors have interdependent roles in patterning the Drosophila oocyte.. Curr Biol.

[14] Lei Y, Warrior R (2000). The Drosophila Lissencephaly1 (DLis1) gene is required for nuclear migration.. Dev Biol.

[15] Delanoue R, Davis I (2005). Dynein anchors its mRNA cargo after apical transport in the Drosophila blastoderm embryo.. Cell.

[16] Wilkie GS, Davis I (2001). Drosophila wingless and pair-rule transcripts localize apically by dynein-mediated transport of RNA particles.. Cell.

[17] Bullock SL, Ish-Horowicz D (2001). Conserved signals and machinery for RNA transport in Drosophila oogenesis and embryogenesis.. Nature.

[18] Tomlinson A (1985). The cellular dynamics of pattern formation in the eye of Drosophila.. J Embryol Exp Morphol.

[19] Whited JL, Cassell A, Brouillette M, Garrity PA (2004). Dynactin is required to maintain nuclear position within postmitotic Drosophila photoreceptor neurons.. Development.

[20] Ran B, Bopp R, Suter B (1994). Null alleles reveal novel requirements for Bic-D during Drosophila oogenesis and zygotic development.. Development.

[21] Paré C, Suter B (2000). Subcellular localization of Bic-D::GFP is linked to an asymmetric oocyte nucleus.. J Cell Sci.

[22] Nakajima H, Toyoshima-Morimoto F, Taniguchi E, Nishida E (2003). Identification of a consensus motif for Plk (Polo-like kinase) phosphorylation reveals Myt1 as a Plk1 substrate.. J Biol Chem.

[23] Schriefer LA, Waterson RH (1989). Phosphorylation of the N-terminal region of Caenorhabditis elegans paramyosin.. J Mol Biol.

[24] Suter B, Romberg LM, Steward R (1989). Bicaudal-D, a Drosophila gene involved in developmental asymmetry: localized transcript accumulation in ovaries and sequence similarity to myosin heavy chain tail domains.. Genes Dev.

[25] Mohler J, Wieschaus EF (1986). Dominant maternal-effect mutations of Drosophila melanogaster causing the production of double-abdomen embryos.. Genetics.

[26] Ephrussi A, Dickinson LK, Lehmann R (1991). Oskar organizes the germ plasm and directs localization of the posterior determinant nanos.. Cell.

[27] Kim-Ha J, Smith JL, Macdonald PM (1991). oskar mRNA is localized to the posterior pole of the Drosophila oocyte.. Cell.

[28] Daub H, Olsen JV, Bairlein M, Gnad F, Oppermann FS (2008). Kinase-selective enrichment enables quantitative phosphoproteomics of the kinome across the cell cycle.. Mol Cell.

[29] Dephoure N, Zhou C, Villén J, Beausoleil SA, Bakalarski CE (2008). A quantitative atlas of mitotic phosphorylation.. Proc Natl Acad Sci U S A.

[30] Bodenmiller B, Mueller LN, Pedrioli PG, Pflieger D, Jünger MA (2007). An integrated chemical, mass spectrometric and computational strategy for (quantitative) phosphoproteomics: application to Drosophila melanogaster Kc167 cells.. Mol Biosyst.

[31] Zhai B, Villén J, Beausoleil SA, Mintseris J, Gygi SP (2008). Phosphoproteome analysis of Drosophila melanogaster embryos.. J Proteome Res.

[32] Blom N, Gammeltoft S, Brunak S (1999). Sequence and structure-based prediction of eukaryotic protein phosphorylation sites.. J Mol Biol.

[33] Obenauer JC, Cantley LC, Yaffe MB (2003). Scansite 2.0: Proteome-wide prediction of cell signaling interactions using short sequence motifs.. Nucleic Acids Res.

[34] Frame S, Cohen P (2001). GSK3 takes centre stage more than 20 years after its discovery.. Biochem J.

[35] Oh J, Baksa K, Steward R (2000). Functional domains of the Drosophila bicaudal-D protein.. Genetics.

[36] Bellen HJ, Levis RW, Liao G, He Y, Carlson JW (2004). The BDGP gene disruption project: single transposon insertions associated with 40% of Drosophila genes.. Genetics.

[37] Bischof J, Maeda RK, Hediger M, Karch F, Basler K (2007). An optimized transgenesis system for Drosophila using germ-line-specific {varphi}C31 integrases.. Proc Natl Acad Sci U S A.

[38] Rørth P (1998). Gal4 in the Drosophila female germline.. Mech Dev.

[39] Arya R, Lakhotia SC (2006). A simple nail polish imprint technique for examination of external morphology of Drosophila eyes.. Curr Sci.

[40] Stern DL, Sucena E, Sullivan W, Ashburner M, Hawley RS (2000). Preparation of Larval and Adult Cuticles for Light Microscopy.. Drosophila Protocols.

[41] Mancebo R, Zhou X, Shillinglaw W, Henzel W, Macdonald PM (2001). BSF binds specifically to the bicoid mRNA 3′ untranslated region and contributes to stabilization of bicoid mRNA.. Mol Cell Biol.

[42] Claußen M, Koch R, Jin ZY, Suter B (2006). Functional characterization of Drosophila Translin and Trax.. Genetics.

[43] Delorenzi M, Speed T (2002). An HMM model for coiled-coil domains and a comparison with PSSM-based predictions.. Bioinformatics.

[44] Notredame C, Higgins DG, Heringa J (2000). T-Coffee: A novel method for fast and accurate multiple sequence alignment.. J Mol Biol.

