# Supplementary material for: Systematic Functional Analysis of Bicaudal-D Serine Phosphorylation and Intragenic Suppression of a Female Sterile Allele of BicD
Source: PLoS One. 2009 Feb 23;4(2):e4552. doi: 10.1371/journal.pone.0004552 (PMC2639643; doi:10.1371/journal.pone.0004552)
Supplement: Figure S1 — Effect of amino acid 103 on BicD localization (0.57 MB PDF) [file pone.0004552.s001.pdf]

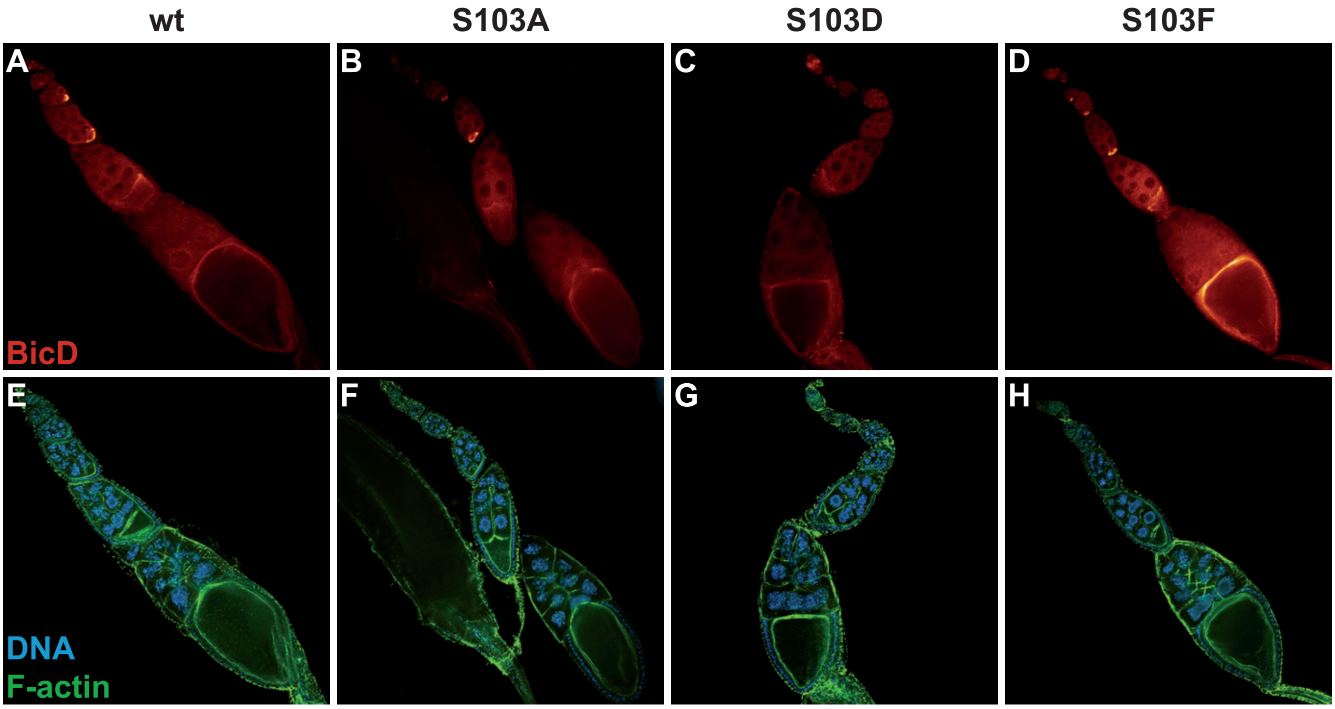

SUPPORTING FIGURE S1. **Effect of amino acid 103 on BicD localization**. Immunostainings of ovaries from indicated BicD mutant females stained with anti-BicD antibodies (red). DNA was visualized with Hoechst (blue), and actin filaments were stained with FITC-phalloidine (green). The intensity of BicD accumulation in the oocyte is dependent of the amino acid 103. Maximum projections of z-stacks are shown. Panels E-H: for clarity reasons, individual panels are composed of different optical sections.
